# Preparation of Glass Fiber Reinforced Polypropylene Bending Plate and Its Long-Term Performance Exposed in Alkaline Solution Environment

**DOI:** 10.3390/polym17131844

**Published:** 2025-06-30

**Authors:** Zhan Peng, Anji Wang, Chen Wang, Chenggao Li

**Affiliations:** 1 Key Lab of Structures Dynamic Behavior and Control, Ministry of Education, Harbin Institute of Technology, Harbin 150090, China; 15941629636@163.com (Z.P.); 17709860030@163.com (A.W.); lichenggao@hit.edu.cn (C.L.); 2 Key Lab of Smart Prevention and Mitigation of Civil Engineering Disasters, Ministry of Industry and Information Technology, Harbin Institute of Technology, Harbin 150090, China; 3 School of Civil Engineering, Harbin Institute of Technology, Harbin 150090, China

**Keywords:** glass fiber reinforced polypropylene plate, bending angle, alkali solution immersion, mechanical properties, thermal properties, degradation mechanism

## Abstract

Glass fiber reinforced polypropylene composite plates have gradually attracted more attention because of their repeated molding, higher toughness, higher durability, and fatigue resistance compared to glass fiber reinforced thermosetting composites. In practical engineering applications, composite plates have to undergo bending effect at different angles in corrosive environment of concrete, including bending bars from 0~90°, and stirrups of 90°, which may lead to long-term performance degradation. Therefore, it is important to evaluate the long-term performance of glass fiber reinforced polypropylene composite bending plates in an alkali environment. In the current paper, a new bending device is developed to prepare glass fiber reinforced polypropylene bending plates with the bending angles of 60° and 90°. It should be pointed out that the above two bending angles are simulated typical bending bars and stirrups, respectively. The plate is immersed in the alkali solution environment for up to 90 days for long-term exposure. Mechanical properties (tensile properties and shear properties), thermal properties (dynamic mechanical properties and thermogravimetric analysis) and micro-morphology analysis (surface morphology analysis) were systematically designed to evaluate the influence mechanism of bending angle and alkali solution immersion on the long-term mechanical properties. The results show the bending effect leads to the continuous failure of fibers, and the outer fibers break under tension, and the inner fibers buckle under compression, resulting in debonding of the fiber–matrix interface. Alkali solution (OH^−^ ions) corrode the surface of glass fiber to form soluble silicate, which is proved by the mass fraction of glass fiber decreased obviously from 79.9% to 73.65% from thermogravimetric analysis. This contributes to the highest degradation ratio of tensile strength was 71.6% (60° bending) and 65.6% (90° bending), respectively, compared to the plate with bending angles of 0°. A high curvature bending angle (such as 90°) leads to local buckling of fibers and plastic deformation of the matrix, forming microcracks and fiber–resin interface bonding at the bending area, which accelerates the chemical erosion and debonding process in the interface area, bringing about an additional maximum 10.56% degradation rate of the shear strength. In addition, the alkali immersion leads to the obvious degradation of storage modulus and thermal decomposition temperature of composite plate. Compared with the other works on the long-term mechanical properties of glass fiber reinforced polypropylene, it can be found that the long-term performance of glass fiber reinforced polypropylene composites is controlled by the corrosive media type, bending angle and immersion time. The research results will provide durability data for glass fiber reinforced polypropylene composites used in concrete as stirrups.

## 1. Introduction

Although traditional reinforced concrete structures have the characteristics of a wide range of raw materials and good overall integrity, they have long been plagued by four main problems: durability defects, high dependence on construction quality, large self-weight, and high energy consumption and emissions. In marine or high chloride ion environments, steel corrosion can cause concrete cracking, leading to a significant reduction in the service life of the structure [1,2]. For example, severe corrosion of docks occurs in southern China within 15 years. The complex process of binding steel bars and pouring concrete can easily lead to insufficient strength or cracks. Steel reinforcements in concrete tend to corrode and this process can lead to structural damage [3,4]. High self-weight significantly increases foundation load and engineering costs. In addition, the high carbon emissions from cement production are even more contrary to sustainable development goals [5]. To break through the dilemma, fiber reinforced polymer (FRP) composite materials have become an ideal alternative material for solving general steel corrosion inside the concrete, reducing self-weight, improving construction efficiency, and reducing carbon footprint due to their disruptive advantages [6,7,8], such as lightweight and high strength, corrosion resistance and maintenance free and convenient construction [9,10]. They have promoted the coordinated development of civil engineering towards long lifespan, low carbonization, and high efficiency in fields, such as bridge strengthening and ocean engineering [11,12].

According to the type of resin matrix, composites can be divided into thermosetting resin and thermoplastic resin composites. Thermosetting resin composite materials (such as epoxy) have inherent defects due to chemical cross-linking of molecular chains [13]. High brittleness leads to easy fracture under impact, interface debonding and hydrolysis under hygrothermal environments, resulting in insufficient durability, weak fatigue resistance under cyclic loading and recycling difficulties [14,15,16], seriously restricting their long-term service reliability. In contrast, thermoplastic resin composites achieve breakthrough innovations through physical entanglement of molecular chain structures [17]. Their repeatable melt forming characteristics support closed-loop recycling, significantly reducing environmental burden. The molecular chain slip mechanism endows excellent toughness and impact resistance, effectively suppressing crack propagation [18,19]. At the same time, non-crosslinked structures have higher stability in hygrothermal environments. Among more thermoplastic composites, glass fiber reinforced polypropylene composite materials (GFRPP) are particularly outstanding [20,21,22]. For example, glass fiber reinforced thermoplastic composites have higher elongation at break and a lower price compared to carbon fiber reinforced thermoplastic composites. At the same time, the polypropylene matrix and glass fibers work together to form a lightweight and high-strength system, which has excellent chemical corrosion resistance and fatigue resistance. It not only avoids the inherent defects of thermosetting materials but also becomes the key solution for promoting engineering material upgrades with high performance, sustainability, and economy compared to carbon fiber reinforced polymer composites [23,24,25,26].

When glass fiber reinforced polypropylene composites plates are used as stirrups inside concrete [27], the bending angle and the long-term effects of the concrete’s alkaline environment may cause the damage and destruction to the composite materials, such as resin cracking, glass fiber erosion, and fiber–resin interface debonding [28,29,30]. Their long-term performance is affected by the coupling effect of pre-damage induced by bending angle and concrete alkali environment erosion. In terms of bending technology, there is relatively little research on the bending process and forming methods of thermoplastic composite, and the bending devices suitable for traditional steel bars are mainly used. For example, Wang et al. [31] achieved a bending effect through the process of positioning the reinforcement→heating softening→bending forming→water tank cooling→reinforcement shaping. When the ratio of bending radius to bar diameter was 2.14 and 2.86, respectively, the tensile strength retention rate of the bending bar was only 26% and 28%, respectively. Lu et al. [32] designed a specialized bending device for thermoplastic epoxy resin composite bar. When the bending radius to bar diameter ratio was 3, 4, and 5, the strength retention rates were only 29%, 30%, and 33%, respectively. Andreas Apitz et al. [33] used a method of heating the bending area and applying force using pulleys to bend carbon fiber reinforced thermoplastic composite bars. Due to the cross-sectional shape of the bars changing from circular to flat strips during the bending process, the final strength retention rate was only 30% to 40%. Thanongsak Imjai et al. [34] reports on an investigation into the effect of section geometry and bond, which led to a new macro-mechanical model to calculate the bend capacity of FRP bars. The results indicated that, compared with existing equations, the proposed model predicts the bend strength of bars more accurately, with an average prediction to experiment ratio of 1:0 and a standard deviation of 0:25. The proposed model can lead to more economical design, by up to 15%. The above analysis shows that the bending process is complex and plays a key role in the mechanical properties of materials. In response to the alkaline environment, scholars have conducted some research on the degradation mechanism. For example, Zhou et al. [35] evaluated the durability of glass fiber reinforced polypropylene (GFRPP) by studying its interlaminar shear strength (ILSS) in distilled water (DW), alkaline solution (AS), and simulated marine concrete environments. The results indicate that after soaking at 60 °C for 120 days, the ILSS retention rates in DW and AS were 67.0% and 67.5%, respectively. The hygrothermal environment accelerates the development of micropores and cracks caused by the extrusion process, and triggers fiber–resin debonding, which is the main cause of mechanical performance degradation. The life prediction model based on Arrhenius acceleration theory shows that the service life of GFRPP reinforcement in AS environment is significantly shorter than that in DW environment due to the destructive effect of glass fiber dissolution. Xian et al. [36] conducted an accelerated aging experiment using an alkaline solution for 180 days, revealing the long-term performance degradation mechanism. Bending bars have a higher water absorption rate than straight bars due to processing microcracks, but a lower diffusion rate. Alkali erosion causes glass fiber dissolution (mass loss) and interface debonding, resulting in a decrease in the retention rate of flexural strength to 20.6–56.0%, and the failure mode changes from fiber fracture to shear fracture. The degradation mechanisms include glass fiber dissolution, interface failure, and increased porosity (about 23.7%). Based on the degradation model prediction, the service life of GFRPP flexural bars in different regions ranges from 1.7 years (Haikou) to 3.9 years (Harbin), with a corresponding strength retention rate of 22.13%. Benmokrane et al. [37] compared the physical and mechanical properties of thermoplastic and thermosetting GFRP bar and evaluated their long-term durability through alkaline environment simulation. The results indicate that the mechanical properties of thermoplastic GFRP bar are excellent with the tensile strength of 1421 MPa (thermoplastic GFRP bar) and meet the standards (1000 MPa) of high-grade GFRP bar (CSA-S807 2019). The retention of tensile strength after alkaline exposure is 87%, and the retention rate of modulus is 100%. The creep strain sustained for 10,000 h under a 40% ultimate tensile strength (UTS) load is only 8% of the initial value. This material combines processability and high durability, providing a new corrosion resistance enhancement solution for concrete structures. Zhang et al. [38] developed a new type of glass fiber reinforced polypropylene (GFRPP) self-anchored plate cable, which utilizes the high toughness/durability of thermoplastic resin and self-anchored bearing system to solve the reliability problem of composite anchoring. Combined with alkali solution immersion experiments and thermogravimetric/morphological analysis, the long-term performance evolution mechanism is revealed. The results indicate that the composite reinforcement process effectively avoids delamination failure in the transition zone of the plate and cable, as well as interlayer cracking in the straight zone. The retention rate of the tensile strength of the plate cable decreases from 45.6% to 34.0% due to the increased stress concentration in the arc transition zone caused by the large angle. After immersing in alkaline solution, the minimum retention rate of strength decreased to 29.7%, and the main degradation factors were glass fiber dissolution, pore formation, and fiber/resin interface debonding. To summarize, it can be seen that the existing research work on the durability of thermoplastic composites ignores the effect of bending angle. At the same time, the mechanism of performance degradation caused by the coupling effect of alkali solution and bending angle is still unclear.

In the current paper, a new bending device is developed to prepare glass fiber reinforced polypropylene composite bending plate with the bending angles of 60° and 90°. The influence mechanism of bending angle on the tensile properties of plate was systematically evaluated. Subsequently, the plate is immersed in an alkali solution environment for up to 90 days for long-term exposure. Mechanical properties, thermal properties, and micro-morphology analysis were systematically designed to evaluate the influence mechanism of alkali solution immersion and bending angle on the long-term mechanical properties of plate. The research results will provide durability data for glass fiber reinforced polypropylene composites used in concrete as stirrups.

## 2. Experimental Program

### 2.1. Raw Materials

The raw material used in this paper is bidirectional glass fiber reinforced polypropylene composite plate, which comes from Shandong Tuoyan New Material Technology Co., Ltd. (Zaozhuang, China). It should be noted that the tacticity of polypropylene adopted in this paper is isotactic with the molecular weight range of 200,000–600,000 g/mol. The above composite plate was prepared by molding process with a thickness of 1.6 mm, a width of 25 mm and a density of 3500 g/m^2^ [39]. The volume fractions of glass fiber and resins are about 70% and 30%, respectively.

### 2.2. Preparation Technology of Bending Plate

#### 2.2.1. Design Idea and Equipment Design

The bending method of GFRPP plate uses the principle of heating–melting and cooling molding of thermoplastic resin, heats the bending part of the plate to above the melting point temperature of the resin, and obtains a bending plate with a certain angle after the resin temperature is cooled to room temperature. The GFRPP plate bending device includes bending disc, baffle, rubidium magnet, and other components. The bending process includes two parts: bending equipment installation and bending experiment. In the bending test, firstly, the heating belt is selected at the bending point of the GFRP plate and attached to the baffle. GFRPP plate is then clamped with magnets. Then, heating the plate in the bending area, controlling the heating temperature by a voltage regulator, leveling the plate in the bending area by a roller, and clamping it again by a rubidium magnet after the plate in the bending area is softened manually to a certain bending angle. Finally, after the temperature of the bending zone drops to room temperature, the bending equipment is removed and the bending plate is removed.

The features of the bending equipment include the following: (1) The central column can be processed with multiple outer diameters according to the needs of use to obtain GFRP bending plates with different bending radii of curvature. The heating device is wrapped with an inner layer of tin foil and wrapped with an outer layer of heating belt, which can ensure that the GFRP plates are heated more evenly. (2) The winding of the heating belt has a certain restraint effect on the bending part, effectively limiting the deformation of the bending section during the bending process. In addition, the heating temperature is accurately controlled by voltage regulators and thermocouples, which can make GFRP plates soften. The inner layer wrapped with tin foil can effectively avoid direct contact with the heating belt after the resin softens. (3) The smooth baffle can effectively limit the cross-sectional deformation of GFRP plate during the rotation process and protect the plate in the bending area. At the same time, the groove diameter should be determined according to the diameter and thickness of GFRP plate. (4) The device has strong flexibility and can realize bending of GFRP plates with different diameters with different curvature radii and angles. The surface of the bending disc is marked with a scale, which can bend at different bending angles. According to the scale on the bending disc, it can be turned to the desired angle and stopped. After the expected angle is reached, it can be fixed and cooled.

#### 2.2.2. Preparation Process of Bending Plate

**Figure 1:**
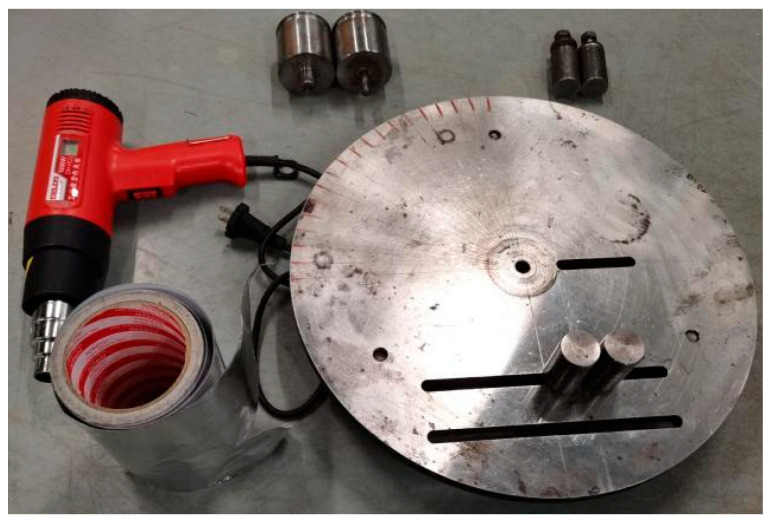
Schematic diagram of bending fixture of glass fiber reinforced polypropylene plate.

**Figure 2:**
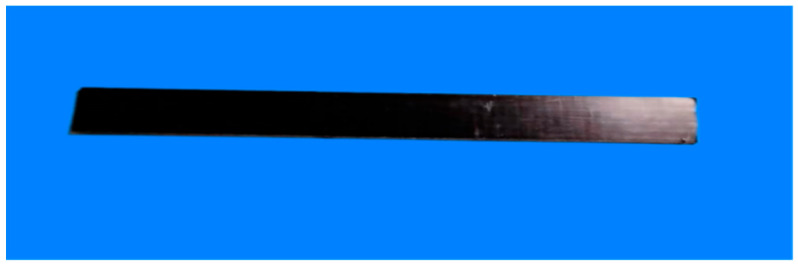
Glass fiber reinforced polypropylene composite plate.

**Figure 3:**
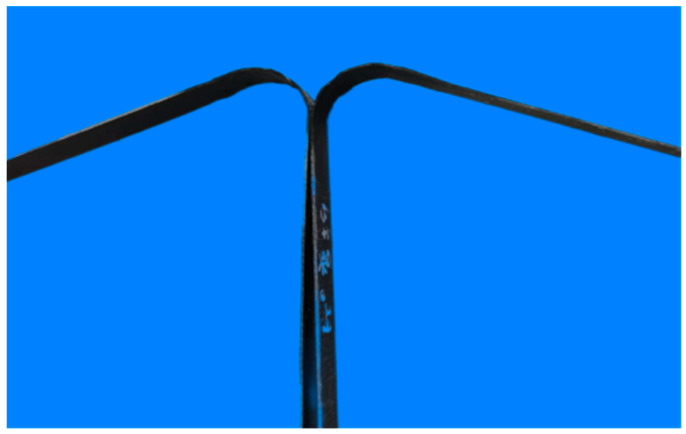
Glass fiber reinforced polypropylene composite bending plate with a bending angle of 60 degrees.

Firstly, the alcohol is used to clean the impurities of the bending disc, baffle, roller, and rubidium magnet. After cleaning, put the above parts for later use. All device components are shown in Figure 1. Then, the thermoplastic composite plate is cut into long strips along the fiber direction by manual measurement and cutting to obtain the strip material in Figure 2. The heating device is wrapped with an inner layer of tin foil and wrapped with an outer layer of heating belt, which can ensure that the GFRPP plate is heated more evenly. At the same time, the winding of the heating belt has a certain restraint effect on the bending part, which effectively limits the deformation of the bending section during bending. In addition, the heating temperature is accurately controlled to 180 °C by voltage regulators and thermocouples, which is higher than melting temperatures for GFRPP plates (~170 °C). In addition, the heating temperature should not be too high to excessive resin flow of the bending plate. The inner layer wrapped with tin foil can effectively avoid direct contact with the heating belt after the resin softens. Figure 3 shows the glass fiber reinforced polypropylene composite bending plate. It should be pointed out that in this paper, three kinds of bending angles are developed with bending angles of 0 degrees, 60 degrees, and 90 degrees, respectively, to study the influence of bending angles on the mechanical properties of plate.

### 2.3. Exposure Condition

To explore the durability of bending plates in concrete environments, the finished bending members were soaked in alkali solution to simulate the concrete pore fluid. The alkali solution was configured according to the American Concrete Institute specification ACI440.3R, and the content of each component was as follows: Ca (OH)_2_: 118.5 g, NaOH: 0.9 g, and KOH: 4.2 g per liter of deionized water [40]. The PH value of the alkali solution obtained from the configuration standard is about 12.6. It should be noted that the PH value of the alkali solution should be adjusted in time during the test to ensure that it maintains a stable PH value and simulates the actual service environment of the plate. The adjustment method is to regularly replenish the solution and test its pH value. Each experiment includes three specimens with temperature conditions of 60 °C, time intervals of 30 days, 60 days, and bending angles of 60 degrees and 90 degrees.

### 2.4. Mechanical Tests

#### 2.4.1. Tensile Tests

The standard adopted in the tensile property test is ASTM D638, the selected equipment is a universal mechanical testing machine, the experimental parameters are set as a tensile rate of 5 mm/min, and the temperature is kept at room temperature for tensile strength test. Tensile tests were carried out on the specimens after bending at different angles using a tensile machine. The special property of thermoplastic resin that can be heated and recovered multiple times can solve the problem of anchoring difficulties. Therefore, for composite plates with different bending angles, secondary heating is carried out to restore them to the shape of straight plates for tensile testing to evaluate the bending effect. Three samples were tested for each condition to obtain the average.

#### 2.4.2. Short Beam Shear Tests

The test standard adopted in the short beam shear test is ASTM D2344, the selected equipment is a universal testing machine (DHY-10080), and the experimental parameters are set to a loading speed of 1.3 mm/min. The transition section of the straight plate and bending plate is selected to carry out the shear performance test. Three samples were tested for each condition to obtain the average.

### 2.5. Dynamic Mechanical Analysis (DMA)

The thermomechanical properties of the thermoplastic bending plate were tested using dynamic thermomechanical analysis (DMA). The test method is to use a single cantilever as the test mode. The test specimen size was 40 mm (length), 20 mm (width), and 1.6 mm (thickness). The installation method of the test specimen is to use a professional wrench to fix the sample to the clamp base. The wrench can apply a certain torque to ensure that the sample to fixed inside the clamp. The test temperature is gradually increased from room temperature to 200 °C at 5 °C/min. The load frequency is 1 Hz. One sample was used for testing.

### 2.6. Thermogravimetric Analysis Tests (TGA)

In order to evaluate the thermal properties of the bending plate before and after the immersions, thermogravimetric analysis tests were conducted through using TGA (NETZSCH STA 449C, Selb, Germany) to obtain the mass variations from room temperature to 800 °C at a heating rate of 10 °C/min in air. The sample weight was about 10 mg and the dry air flow was 20 mL/min. The mass fraction of glass fiber before and after the immersion can be obtained from the thermogravimetric curves. One sample was used for testing.

### 2.7. Scanning Electron Microscopy (SEM)

The surface morphology analyses of the GFRPP plate before and after the immersions were conducted through the scanning electron microscope (SEM, VEGA3, Brno, Czech Republic). The samples were vacuumed and sprayed with gold to increase the electrical conductivity. The gold-coating thickness is 10~15 nm and the chamber vacuum level is 10^−4^~10^−6^ Pa. then, 1000 Hz and 0.7 A were selected as the test frequency and electric current, respectively, and the voltage amplitude was 30 kV. One sample was used for testing.

## 3. Results and Discussion

### 3.1. Tensile Properties of Thermoplastic Bending Plate

**Figure 4:**
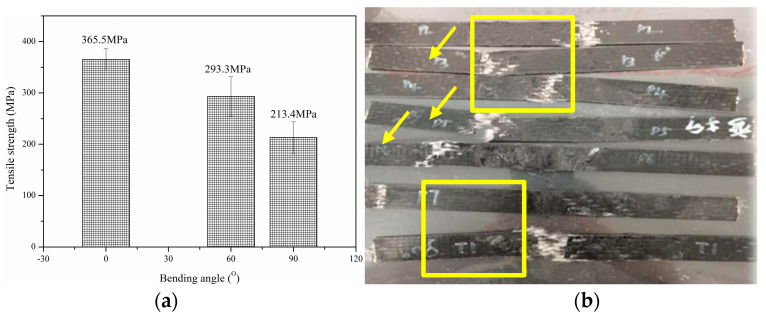
Effect of bending angle on tensile properties of glass fiber reinforced polypropylene composite bending plate. (**a**) Tensile strength, (**b**) failure mode.

Figure 4 shows the effect of bending angle on the tensile properties of thermoplastic bending plate. The effect of bending angle on the tensile strength of thermoplastic composite plates is essentially the process of multi-scale damage accumulation and failure mode transition induced by geometric defects. As shown, with the increase in bending angle from 0° to 90°, the tensile strength decreases significantly (365.5 MPa→213.4 MPa). The degradation mechanism can be attributed to two aspects: (1) Bending leads to the continuous failure of fibers, especially at large angles (90°): the outer fibers break under tension, and the inner fibers buckle under compression, resulting in debonding of the fiber–matrix interface [21]. The geometric abrupt change at the bending apex forms a high stress concentration area, which accelerates the initiation of matrix microcracks. The “transition zone burst” of 60° bending part is the matrix shear failure triggered by stress concentration, while the “surface crack” of 90° bending part is the starting point of fiber fracture and delamination propagation [18]. (2) The increase in bending angle aggravates the irreversible damage of resin matrix: the outer bending matrix produces micro-cracks under tension, and the compression creep of the inner matrix leads to the obstruction of molecular chain rearrangement, forming a high residual strain zone. The non-polar nature of thermoplastic polypropylene resins makes their interfacial bonding weak, and the increase in interfacial shear stress caused by bending leads to debonding. The burst essence of 60° bending is the sudden release of interface failure, while the residual stress superimposed with external loading at 90° bending makes the actual stress far exceed the nominal value, which leads to early-stage delamination. Therefore, the increase in bending angle intensifies fiber fracture, interface debonding and delamination. This can also be further explained by the fact that the plate failure at a 90 degree bending angle is generally in the middle (Figure 4b—rectangular squares). In contrast, the failure mode at a 60 degree bending angle generally appears at both ends (Figure 4b—arrows).

### 3.2. Effect of Alkali Solution Immersion on Mechanical Properties of Thermoplastic Bending Plate

#### 3.2.1. Tensile Properties

**Figure 5:**
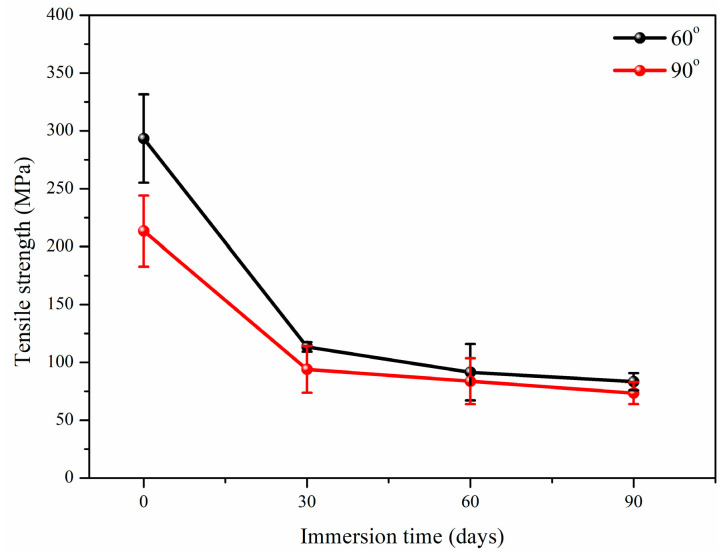
Tensile strength of glass fiber reinforced polypropylene composite bending plate with the immersion.

Figure 5 shows the tensile strength of glass fiber reinforced polypropylene composite bending plate with the immersion time. The tensile strength degradation in alkali environment is the result of the synergistic effect of chemical corrosion and physical stress, and its degradation process is significantly controlled by bending angle. The alkali solution (OH^−^ ions) attacks the material through dual paths: one is to destroy the crystalline region, triggering swelling and microcrack propagation in the amorphous region [41]. The second is to corrode the surface and interface phase of glass fiber, so that the Si-O bond of fiber is dissolved to form soluble silicate. At the same time, alkali solution penetrates into the fiber/matrix interface to cause debonding and weaken the stress transfer efficiency [42].

The bending angle forms a dynamic coupling mechanism with alkali erosion by changing the internal stress distribution and micro-defect morphology. Although the initial deformation of 60° bending is small, a local high stress concentration area is produced at the bending, which preferentially induces the microcrack network. These microcracks acted as rapid penetration channels of alkali solution, accelerating resin plasticization and interface peeling, resulting in more severe initial degradation. With the prolonged immersion, the interfacial reaction produces the accumulation in the narrow bending zone to produce expansion stress, which cooperates with chemical corrosion to make the material show steady and continuous strength attenuation. For a 90° bending plate, with the immersion time, the residual stress accumulated by high plastic deformation continues to be released, which promotes the matrix microcracks to propagate along the fiber direction. At the same time, the alkali solution gradually penetrates into the interface to cause large area debonding, and the bending stress in the high angle region intensifies the dissolution, resulting in the larger degradation rate exceeding 60° bending plate.

#### 3.2.2. Short Beam Shear Strength

**Figure 6:**
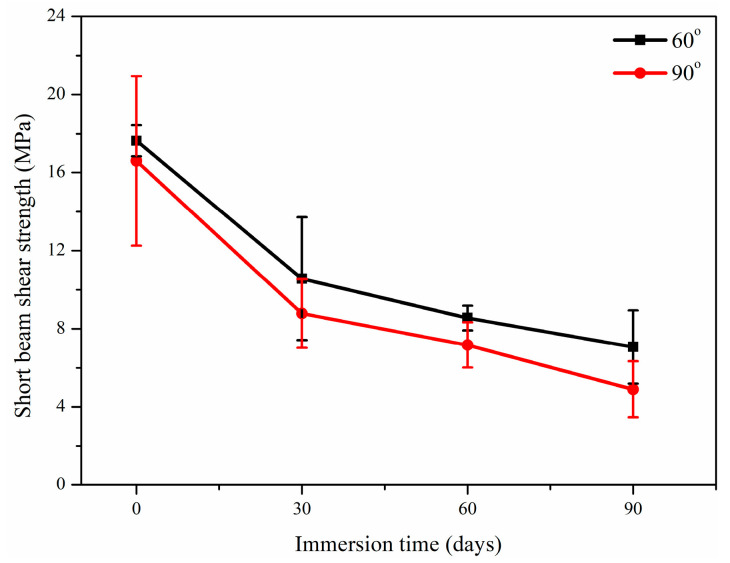
Short beam shear strength of glass fiber reinforced polypropylene composite bending plate with immersion.

Figure 6 shows the variation in short beam shear strength of glass fiber reinforced polypropylene composite bending plate with immersion time. It can be observed that as the immersion time increases, the shear strength of the plate continuously decreases. At immersion time of 30 days, 60 days, and 90 days, the retention rates of the shear strength of the 60 degree bending plate are 59.9%, 48.5%, and 40.1%, respectively; 90 degree bending plate are 52.9%, 43.2%, and 29.5%, respectively. It can be observed that the bending angle has a significant effect on the long-term variation in the shear strength of bending plate, with higher bending angles resulting in lower shear strength retention rates. The main reasons for the degradation of shear strength are attributed to two reasons: the corrosion effect of alkali solution and the residual stress effects from bending angle [43].

The degradation mechanism of short beam shear strength of glass fiber reinforced polypropylene composite plates with immersion time in alkaline solution environment is interface failure. Alkali penetration leads to plasticization of polypropylene matrix, reducing its strength and ability to wrap fibers [35]. The dissolution of glass fiber surface by alkaline solution and the propagation of interface microcracks further exacerbate the degradation of overall material properties. The influence mechanism of bending angle on long-term performance is mainly reflected in the pre-damage and residual stress. Higher bending angles (such as 90°) cause more significant fiber local buckling/fracture and matrix plastic deformation in the manufacturing process, resulting in greater residual stress and initial microcracks, which directly weakens the material strength and provides a preferential penetration channel for alkali solution, accelerating the degradation of the interface area. Meanwhile, high bending angles can lead to more severe stress concentration, especially at the bending vertices. Combined with the softening and weakening effect of alkali solution on the matrix and interface, delamination and interface shear failure are more likely to occur. In summary, the performance degradation of materials is the result of the synergistic effect of chemical physical erosion (especially interface failure) caused by alkaline solution and geometric mechanical defects (residual stress, pre-damage, stress concentration) introduced by bending process on the immersion time scale. Higher bending angles exacerbate these negative factors, leading to lower long-term strength retention.

#### 3.2.3. Summary of Mechanical Properties

Table 1 summarizes the tensile and shear strength retention of glass fiber reinforced polypropylene bending plate after the immersion. It can be found that with the increase in immersion time, the tensile strength and short shear strength obviously decrease. At the same time, the bending angle has a significant influence on the mechanical properties, and the higher bending angle leads to the lower strength, such as after 90 days of immersion, the retention rates of tensile strength were 28.4% (60° bending angle) and 34.4% (90° bending angle), respectively, and the retention rates of short beam shear strength were 40.08% (60° bending angle) and 29.52% (90° bending angle), respectively. The degradation mechanism of the bending plate stems from the interface failure caused by the synergistic effect of alkali erosion and bending angle. Alkali solution breaks the chemical bonds of glass fiber (Si-O bond), causing fiber to matrix debonding and directly weakening stress transfer efficiency. The bending angle amplifies this degradation process through geometric and mechanical effects. A high bending angle (90°) causes local fiber buckling and matrix plastic deformation during manufacturing, forming residual stress concentration zones and initial microcracks (such as at the bending vertex), significantly accelerating the penetration of alkali solution along the interface. At the same time, high curvature leads to an increase in fiber–resin interface bonding, making the bending area more prone to delamination and shear slip under stress. Coupled with the erosion effect of alkali solution on the interface, the strength retention rate of high bending angle plates is ultimately lower; for example, the shear strength retention rate of 29.52% for 90° bending is significantly lower than that of 40.08% for 60° bending. Therefore, the essence of performance degradation is the coupling effect of chemical interface erosion and bending structural defects in the time and stress dimensions.

### 3.3. Long-Term Evolution of Thermal Properties

#### 3.3.1. Dynamic Mechanical Analysis

**Figure 7:**
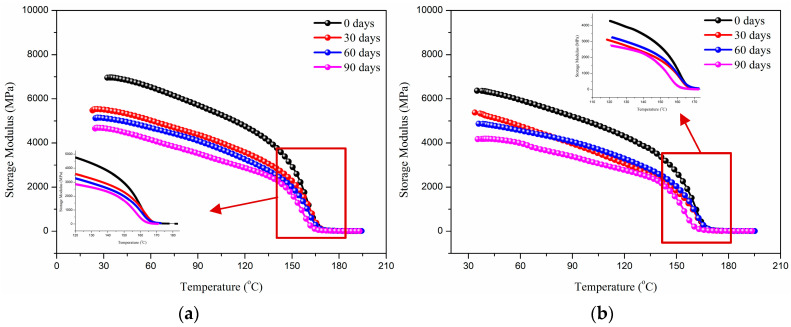
Storage modulus of glass fiber reinforced polypropylene composite bending plate with the immersion time. (**a**) Bending angle of 60°, (**b**) bending angle of 90°.

After the immersion, the storage modulus of glass fiber reinforced polypropylene (GFRPP) composite plates is shown in Figure 7. As shown, it gradually decreases with increasing immersion time, and the increase in bending angle from 60° to 90° further exacerbates this phenomenon. This is because the synergistic effect of interface failure caused by alkali corrosion and stress damage caused by bending structure. The OH^−^ ions in the alkaline solution continuously attack the Si-O bonds on the surface of the glass fiber, causing the breakage of the silicon oxygen network, weakening the bonding strength of the fiber–resin interface, resulting in a decrease in stress transfer efficiency and directly leading to a decrease in storage modulus [44]. The increase in bending angle introduces higher residual stress and pre-damage defects inside the material. High curvature bending leads to local buckling of fibers and plastic deformation of the matrix, forming microcracks and fiber–resin interface bonding at the bending area. This not only directly weakens the local stiffness of the material, but also provides a fast channel for alkali penetration, accelerating the chemical erosion and debonding process in the interface area. At the same time, the geometric stress concentration effect in the bending area further amplifies the interface delamination, resulting in a significant decline in the elastic recovery ability (i.e., storage modulus) of the material. Therefore, the decrease in storage modulus is the result of the coupling effect of chemical interface degradation (alkaline erosion weakens the fiber and interface) and mechanical pre-damage (bending angle exacerbates defects and stress concentration).

**Figure 8:**
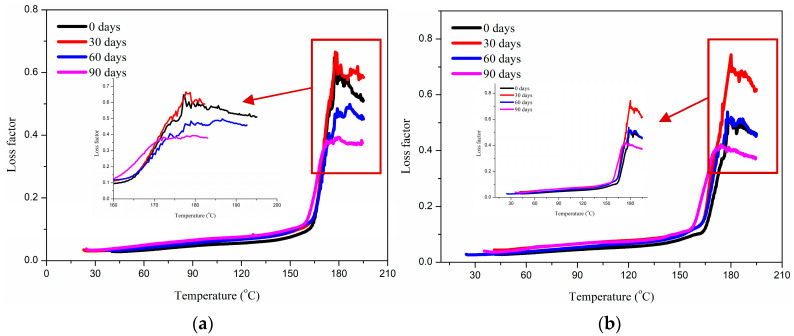
Loss factor of glass fiber reinforced polypropylene composite bending plate with immersion time. (**a**) Bending angle of 60°, (**b**) bending angle of 90°.

Figure 8 shows the loss factor of glass fiber reinforced polypropylene composite bending plate with the immersion time. The peak value of the loss factor (tan δ) of glass fiber reinforced polypropylene (GFRPP) bending plates firstly increases and decreases with immersion time in alkaline solution environment, indicating the critical transition of the material from plasticizing energy dissipation to brittle degradation. In the initial stage (0–30 days), alkaline infiltration leads to plasticization of the polypropylene matrix and micro-debonding at the interface, and the increase in the degree of freedom of molecular chain movement causes an increase in tan δ peak value. After long-term immersion (30~90 days), the matrix breaks due to excessive hydrolysis of resin molecular chains, forming rigid short chains and losing viscoelasticity. At the same time, the SiO_2_ network on the surface of the glass fiber was deeply dissolved by alkaline solution (Si-O-Si bond fracture). The fiber–resin interface completely lost its energy dissipation ability, leading to the decrease in tan δ peak value. 

On the other hand, it can be found that the melting temperature of glass fiber reinforced polypropylene composites gradually decreases with the increase in immersion time. The gradual decrease in melting temperature of glass fiber reinforced polypropylene (GFRPP) composite materials in an alkaline solution environment is essentially the result of the synergistic effect of molecular chain breakage and crystal defects. The active ions (such as OH^−^) in the alkaline solution continues to attack the weak sites in the polypropylene molecular chain, causing hydrolysis and chain breakage reactions, resulting in a significant decrease in molecular weight. The short molecular segments generated by chain breakage not only disrupt the regularity of the molecular chain but also act as “impurities” embedded in the crystal structure, hindering the formation of a perfect crystal. The above effect reduces the energy required for the material to melt, ultimately resulting in a systematic decrease in melting point temperature with immersion time.

#### 3.3.2. Thermogravimetric Analysis of Glass Fiber Reinforced Polypropylene Bending Plate

**Figure 9:**
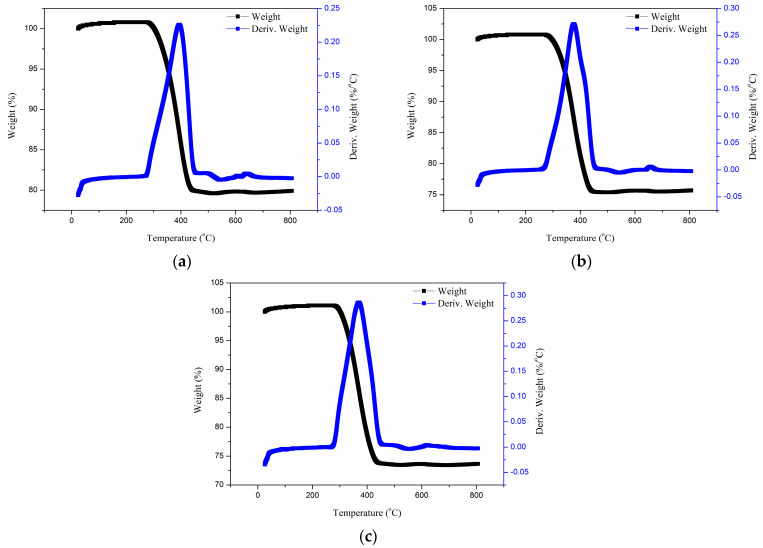
Weight loss curve and its first derivative of glass fiber reinforced polypropylene composite bending plate with different bending angles. (**a**) Control (before the immersion), (**b**) 60°–90 days, (**c**) 90°–90 days.

Figure 9 shows the weight loss curve and its first derivative of a glass fiber reinforced polypropylene composite bending plate with different bending angles. It can be found that after immersion in alkali solution, the mass fraction of the glass fiber decreased obviously from 79.9% to 75.68% (60° bending plate) and 73.65% (90° bending plate). At the same time, the maximum thermal decomposition temperature also decreased from 394.6 °C to 375.69 °C (60° bending plate) and 369.08 °C (90° bending plate). The OH^−^ ions in alkaline solution continuously corrode the silicon oxygen network on the surface of glass fibers (Si-O-Si bond breakage), causing fiber dissolution and the release of soluble silicates, directly reducing the proportion of fiber mass. High curvature bending leads to local fiber buckling and interface debonding, forming stress concentration zones and microcrack networks at the bending vertices. This not only directly exposes more fiber surfaces to alkaline erosion but also widens the channels for corrosive media penetration, deepening the degree of fiber dissolution. In addition, the matrix pre-damage caused by bending (plastic deformation and micropores) further reduces the thermal stability of the matrix [45], combined with the erosion effect of fiber interface degradation products, leading to a systematic decrease in the overall thermal decomposition temperature of the material. Therefore, the synchronous decline of fiber mass fraction and thermal stability is essentially a chain coupling effect of chemical dissolution (fiber dissolution matrix chain breakage) and mechanical damage (bending stress amplification corrosion). Higher bending angles strengthen these two mechanisms, resulting in more severe performance degradation.

### 3.4. Degradation Mechanism Analysis

**Figure 10:**
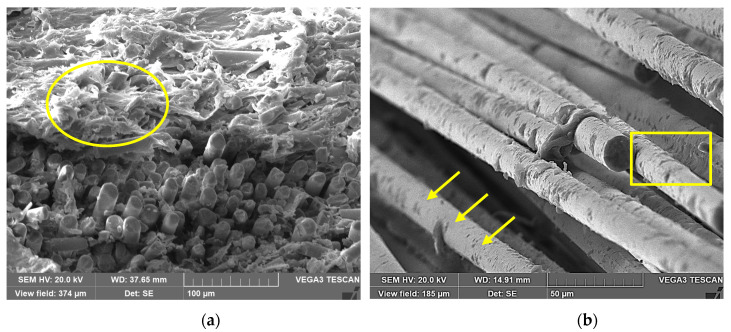
Surface morphology analysis of glass fiber reinforced polypropylene bending plate before and after immersion. (**a**) Control (before the immersion), (**b**) 60°–3 M.

Based on the observation results of scanning electron microscopy (SEM) in Figure 10, the degradation mechanism of the glass fiber reinforced polypropylene (GFRPP) composite bending plate after immersion in alkaline solution can be attributed to a chain failure process of interface corrosion and fiber dissolution. The polypropylene resin uniformly and densely wraps the glass fibers in the original sample, forming a tight interface bond, SEM shows no exposed fiber surface, and the interface transition zone is continuous, endowing the material with excellent mechanical properties. After three months of immersion in alkaline solution, SEM images clearly revealed dual degradation characteristics: (1) Dissolution of the glass fiber surface: OH^−^ ions in the alkaline solution continued to attack the silicon oxygen network (Si-O-Si bonds) on the fiber surface, resulting in groove like etching and micropores on the fiber surface, directly weakening the fiber’s own strength; (2) Interface debonding dominant failure: alkaline solution penetrates into the fiber resin interface, causing a sharp drop in interfacial bonding force. The synergistic effect of fiber damage and interface adhesion loss not only directly explains the significant degradation of material mechanical properties (such as strength and modulus)—interface debonding prevents effective load transfer from the matrix to the fibers but also reveals the root cause of thermal performance degradation.

### 3.5. Comparison with Others’ Work

After obtaining the long-term degradation mechanism of glass fiber reinforced polypropylene bending plate, Table 2 conducts the comparison and analysis of the degradation of long-term mechanical properties of glass fiber reinforced polypropylene bending composites exposed to different service environments at 60 °C. The difference in the degradation of tensile and shear properties of glass fiber reinforced polypropylene composites in distilled water and alkaline solution environments is essentially due to the multi-level synergistic mechanism of solution corrosiveness, bending stress, and exposure time. Distilled water only causes matrix plasticization and slight interface swelling through physical penetration. In contrast, OH^−^ ions in alkaline solutions actively attack the Si-O-Si network on the surface of glass fibers, leading to the dissolution of the fiber and the release of soluble silicates. This significantly weakens the stress transfer efficiency of the fiber–resin interface, resulting in lower long-term performance retention of materials in alkaline solutions. The bending angle increases the degradation effect by geometric defects and residual stress amplification. For the straight bars/plate with the 0° bending angle, it has complete interface bonding, uniform stress distribution, and optimal performance retention. When the bending angle increases (such as 60° to 90°), high curvature forming causes local fiber buckling, matrix microcracks, and interface debonding. A stress concentration core area is formed at the bending area, which not only directly reduces the local bearing capacity, but also provides a fast penetration channel for alkaline solution, accelerating interface chemical erosion and delamination expansion, making the 90° bending part with the most significant performance degradation after long-term exposure. The role of exposure time lies in the irreversibility of cumulative damage. With prolonged immersion, the degradation dominated by matrix plasticization in distilled water environment tends to saturate, while the continuous fiber dissolution and interface chemical bond fracture in alkaline environment leads to the continuous deterioration of performance. And the high bending angle further cooperates with this process, resulting in degradation of tensile and shear properties.

## 4. Conclusions

In the current paper, a new bending device is developed to prepare glass fiber reinforced polypropylene bending plate with the bending angles of 60° and 90°. The influence mechanism of bending angle on the tensile properties of plate was systematically evaluated. Subsequently, the plate is immersed in an alkali solution environment for up to 90 days for long-term exposure. Mechanical properties, thermal properties, and micro-morphology analysis were systematically designed to evaluate the influence mechanism of alkali solution immersion and bending angle on the long-term mechanical properties of plate. The following conclusions can be obtained:

(1) The effect of bending angle on the tensile strength of thermoplastic composite plates is essentially multi-scale damage accumulation and failure mode transition induced by geometric defects. Bending effect leads to the continuous failure of fibers, and the outer fibers break under tension, and the inner fibers buckle under compression, resulting in the debonding of the fiber–matrix interface.
(2) Alkali solution (OH^−^ ions) corrode the surface of glass fiber to form soluble silicate, which is obviously proved by the mass fraction of glass fiber decreased from 79.9% to 73.65%. In addition, alkali solution penetrates into the fiber/matrix interface to cause debonding and weakens the stress transfer efficiency. This contributes to the highest degradation ratio of tensile strength being 71.6% (60° bending) and 65.6% (90° bending), respectively.
(3) High curvature bending (such as 90°) leads to local buckling of fibers and plastic deformation of the matrix, forming microcracks and fiber–resin interface bonding at the bending area. This not only directly weakens the local stiffness of the material, but also provides a fast channel for alkali penetration, accelerating the chemical erosion and debonding process in the interface area, bringing about an additional maximum 10.56% degradation of the shear strength.
(4) The alkali immersion process leads to the obvious degradation of storage modulus and thermal decomposition temperature of the composite plate. SEM images revealed the dual degradation characteristics of dissolution of the glass fiber surface and interface debonding dominant failure. Compared with other works, it can be found that the long-term performance of glass fiber reinforced polypropylene composites is controlled by the corrosive media type, bending angle, and immersion time.

## Figures and Tables

**Table 1:** Tensile and shear strength retention of glass fiber reinforced polypropylene bending plate after the immersion (%).

Immersion Time (Days)	Tensile Strength		Short Beam Shear Strength	
	60° Bending Angle	90° Bending Angle	60° Bending Angle	90° Bending Angle
0	100	100	100	100
30	38.66	44.02	59.92	52.91
60	31.78	39.20	48.49	43.17
90	28.40	34.40	40.08	29.52

**Table 2:** Comparison and analysis of the degradation of long-term mechanical properties of glass fiber reinforced polypropylene bending composites exposed to different service environments at 60 °C.

Material Type	Exposure Medium	Exposure Time (Days)	Tensile Strength Retention (%)	Shear Strength Retention (%)	Reference
Straight bars	Distilled water	0/30/75/120	/	100/88.7/72.6/67.0	[35]
Straight bars	Alkaline	0/30/75/120	/	100/91.3/77.0/67.5	[35]
Bending bars (90° bending)	Alkaline	0/30/105/180	100/29.8/23.7/20.6	/	[36]
Bending plate (without bending strain)	Distilled water	0/90/180	100/68.68/58.32	100/70.22/61.65	[39]
Bending plate (low bending strain)	Distilled water	0/90/180	100/59.76/55.57	/	[39]
Bending plate (high bending strain)	Distilled water	0/90/180	100/51.72/46.11	/	[39]
Bending plate cable	Alkaline	0/7/14/21	100/60.0/39.6/29.7	100/75.5/71.5/69.2	[38]
Bending plate (60° bending)	Alkaline	0/30/60/90	100/38.66/31.18/28.40	100/59.92/48.49/40.08	This paper
Bending plate (90° bending)	Alkaline	0/30/60/90	100/44.02/39.20/34.40	100/52.91/43.17/29.52	This paper

## Data Availability

The original contributions presented in this study are included in the article. Further inquiries can be directed to the corresponding authors.
